# Dual HBV cccDNA-linked HiBiT reporter hepatocyte models for screening of candidate cccDNA modulators

**DOI:** 10.1080/21505594.2026.2728526

**Published:** 2026-09-04

**Authors:** Jiali Cao, Yali Zhang, Jian Ma, Shaojuan Wang, Yangtao Wu, Hualong Xiong, Mingfeng Wang, Tianshu Shi, Baorong Fu, Xiaochun Fu, Xiaoli Chen, Huiming Ye, Ningshao Xia, Quan Yuan

**Affiliations:** a Department of Laboratory Medicine, Fujian Key Clinical Specialty of Laboratory Medicine, Women and Children’s Hospital, School of Medicine, Xiamen University, Xiamen, China; b State Key Laboratory of Vaccines for Infectious Diseases, Xiang an Biomedicine Laboratory, School of Public Health, Xiamen University, Xiamen, China

**Keywords:** Hepatitis B virus, cccDNA, reporter model, paired screening, antiviral effect

## Abstract

Chronic hepatitis B remains difficult to cure because the viral covalently closed circular DNA (cccDNA) minichromosome can persist and sustain viral transcription, creating a need for scalable, reporter readouts that facilitate early discovery of cccDNA-modulating agents. Here, we developed two complementary hepatocyte HiBiT reporter models: a replication-competent HBV reporter in HepaRG cells (HepaRG-Hibit16), in which a secreted split-NanoLuc HiBiT signal is linked to cccDNA-associated expression, and a Cre/Lox-based recombinant cccDNA (rcccDNA) reporter in HepG2 cells (HepG2-Rccc1a) that rapidly generates rcccDNA with a matched HiBiT readout. Screening of 1,403 FDA-approved compounds across both models identified 13 concordant, non-cytotoxic hits. Palovarotene, a retinoic acid receptor-γ agonist, was selected as an exemplar concordant hit and reduced HBV antigens, HBV DNA, and cccDNA and inhibited HBV infection in multiple hepatocyte-based *in vitro* systems without overt cytotoxicity at the tested concentrations. Together, this dual-reporter strategy supports efficient cross-model triage of candidate cccDNA modulators for subsequent orthogonal validation.

## Introduction

Hepatitis B virus (HBV) infection, particularly chronic HBV infection, stands as a major global public health challenge. Presently, over 292 million individuals worldwide are living with hepatitis B [1,2]. With the widespread promotion of hepatitis B vaccine (HBV vaccine) inoculation, the incidence of new HBV infections among children has decreased. However, there remains a large population of HBV-positive individuals who require antiviral treatment [3,4]. Chronic HBV infection may evolve into chronic hepatitis B (CHB), cirrhosis, and hepatocellular carcinoma (HCC), leading to more than 800,000 deaths annually due to the infection and related diseases. Without treatment, the five-year cumulative incidence of cirrhosis among CHB patients is estimated to be 10–20%, and 2–5% of those with cirrhosis progressing to liver cancer annually [5]. Therefore, timely and effective treatment for chronic HBV carriers is crucial in reducing the prevalence of end-stage liver diseases.

Current first-line therapies – nucleos(t)ide analogs and pegylated interferon – can suppress viral replication and reduce the risk of liver-related complications, yet sustained off-therapy control is uncommon for many patients [6]. One important reason is the intrahepatic persistence of covalently closed circular DNA (cccDNA), a chromatinised minichromosome that can persist despite therapy and continue to serve as the transcriptional template for viral RNAs and antigens [7,8]. Despite extensive development and clinical trials of numerous anti-HBV drugs, definitive evidence of breakthrough efficacy leading to a clinical cure remains elusive [9–11]. Consequently, the urgent development of more effective drugs for chronic hepatitis B treatment is both critical and necessary.

Progress toward cccDNA-directed interventions is constrained by the limited availability of assays that are mechanistically linked to cccDNA activity, quantitative, and scalable for compound screening. Direct cccDNA measurements, including Southern blotting and PCR-based approaches, are informative but time-consuming, technically demanding, and difficult to adapt to large chemical libraries [12]. In response to these challenges, several surrogate reporter systems have therefore been developed. These models employ antigens HBeAg [13], HA [14], HBsAg [15], luciferase GLuc [16], and fluorescent protein GFP [17] as reporter genes for cccDNA, significantly simplifying the detection process and enhancing throughput compared to direct cccDNA detection. Despite these advancements, challenges remain, such as the complexity of synthesizing recombinant cccDNA in prokaryotic cells before transfection, which complicates and elevates the cost of screening. Further simplification of antigen detection methods is required for their application in million-level throughput drug screening. An ideal model would merge the advantages of these strategies, offering stable HBV cccDNA synthesis alongside easily detectable reporter genes.

Split luciferase has the advantage of short fragment coding sequences, efficient and simple detection method meeting the requirements for high-throughput screening [18]. A recent HBV-HiBiT-PreS2 system further demonstrated the value of a short HiBiT tag for monitoring HBV replication and HBsAg/subviral-particle secretion [19]. We aimed to develop a dual-model workflow that leverages HiBiT luminescence as a proxy for cccDNA-driven transcription in two complementary contexts: (i) a replication-linked system that recapitulates cccDNA formation and maintenance in hepatocyte-like cells, and (ii) a rapid Cre/Lox-based rcccDNA reporter for efficient screening and validation. We report HepaRG-Hibit16 and HepG2-Rccc1a as complementary cccDNA-linked and rcccDNA-linked reporter models. We demonstrate their suitability for screening and apply them to a pilot screen of 1,403 FDA-approved compounds. Concordant hits across both models were prioritized to reduce model-specific artifacts, and one exemplar compound, palovarotene, was carried forward for evaluation in additional *in vitro* HBV systems.

## Materials and methods

### Construction of HBV mutant clones

Genotype D HBV was used as the backbone for all reporter constructs. Exogenous sequences were inserted at the positions shown in the figures and Supplementary figures. The insertion of exogenous genes was achieved by amplifying fragments followed by assembly using Gibson Assembly(New England Biolabs, Ipswich, MA, USA, E2611L), or synthesized by General Biosystems Co., Ltd. (Anhui, China) and Sangon Biotech Co., Ltd(Shanghai, China).

### Selection of stable integrated cell line

The stably integrated cell lines involved in this study were created through the PiggyBac transposon system (System Biosciences, Palo Alto, CA, USA, PB514B-1), integrating target genes into the cell genome. The plasmid carrying the target gene was co-transfected with the expression plasmid for the transposase at a 4:1 ratio into the target cells. After 48 hours, cells were selected by puromycin resistance (InvivoGen, San Diego, CA, USA, ant-pr-5), followed by sorting via flow cytometry.

### Luciferase activity detection

HiBiT detection utilized the Nano Glo HiBiT Extracellular Detection System (Promega, Madison, WI, USA, N2421)with the procedure following the instructions provided by the kit.

### *In vitro* screening of compounds

HepaRG-Hibit16 and HepG2-Rccc1a cells were used to screen for inhibitors of HBV cccDNA. HepaRG-HiBiT16 cells were cultured in Williams’ Medium E (Merck Sigma‑Aldrich, St. Louis, Missouri, USA, W1878) supplemented with 10% FBS (Thermo Fisher Scientific, Waltham, MA, USA, 10,099,141), 100 μg/mL streptomycin and 100 U/mL penicillin (Thermo Fisher Scientific, Waltham, MA, USA, 15,140–122), 100 mM glutamine(Thermo Fisher Scientific, Waltham, MA, USA, 25,030–081), 5 μg/mL insulin (Shanghai Basalmedia Technologies Co., Ltd., Shanghai, China, S454T0), 50 μM hydrocortisone (Stemcell Technologies, Vancouver, BC, Canada, 07926) and 2 μg/mL Puromycin (InvivoGen, San Diego, CA, USA, ant-pr-5). HepG2-Rccc1a cells were cultured in DMEM (Thermo Fisher Scientific, Waltham, MA, USA, 12,100–061) supplemented with 10% FBS (Thermo Fisher Scientific, Waltham, MA, USA, 10,099,141), 100 μg/mL streptomycin and 100 U/mL penicillin (Thermo Fisher Scientific, Waltham, MA, USA, 15,140–122), 2 μg/mL Puromycin (InvivoGen, San Diego, CA, USA, ant-pr-5). Both cell types were digested into cell suspensions and added to 96-well cell culture plates. HepaRG-Hibit16 cells began compound treatment one week after plating, with Doxycycline hyclate (Merck Sigma‑Aldrich, St. Louis, Missouri, USA, D9891) induction at the same time. HepG2-Rccc1a, after plating, were first infected with Adenoviruses carrying Cre recombinase (Ad-Cre, kindly provided by Dr. Shengcai Lin’s lab [20]), then began compound treatment 48 hours later, diluting the test compounds to a final concentration of 1 μM in their respective media, treating the cells for 48 hours before replacing with fresh medium containing the compounds, and detecting HiBiT in the supernatant after 96 hours.

### HBV infection

Using HepG2-hNTCP-2B1 cells [21] as an example, cells were digested into cell suspensions and seeded into 24-well cell culture plates at an appropriate density, about 300,000 cells per well, with Doxycycline to induce hNTCP expression, followed by infection after 4 days. At the time of infection, the virus was diluted in medium to an appropriate titer, mixed with 8% PEG8000 at a 1:1 ratio (final concentration of 4% PEG8000), and incubated with the cells for 16–20 hours. Subsequently, the cells were washed three times with PBS to remove residual virus, and the fresh medium was replaced every two days. Cell supernatants or cell lysates were collected for viral antigen or nucleic acid detection 6–8 days post-infection. PHHs (Livo Biotechnology Co., Ltd., Shenzhen, China, LV-PHH001) were infected or treated with candidate compounds the day after plating, with the culture medium supplemented with “5C” to maintain the characteristics and functions of primary hepatocytes [22].

### Detection of HBV-related antigens and nucleic acids

The level of HBeAg in cell supernatants was detected using the HBeAg Test Kit (Wantai BioPharm, Beijing, China, HBV-0396), and the level of HBsAg in cell supernatants was detected using the HBsAg Test Kit (Wantai BioPharm, Beijing, China, HBV-1396). The total HBV DNA level [23] and HBV cccDNA level [24] were quantified by real-time PCR using protocols as previously described. The concentrations of test samples were calculated based on the curve plotted with the CT values of the standards and their corresponding HBV DNA concentrations, or the relative value of HBV cccDNA to mitochondrial DNA was calculated [25]. HBV cccDNA was also detected by Southern blot using DNA extracted by the Modified Hirt procedure [13], following by electrophoresis, membrane transfer, and hybridization as previously described [23]. For cccDNA sequence confirmation, cccDNA-enriched DNA was amplified using primers spanning the rcDNA gap region (nt1823-1806, 5”-CCGGAAAGCTTGAGCTCTTCAAAAAGTTGCATGGTGCTGG-3;‘ nt2812-2832, 5’-GTGGGTCACCATATTCTTGGG-3‘), and the products were analyzed by Sanger sequencing with primers nt1580-1596 (5’-TGCACTTCgCTTCACCT-3‘) and nt2314-2298 (5’-AGGGGCATTTGGTGGTC-3”).

### Immunoprecipitation and HiBiT blotting

To characterize secreted HiBiT-containing products, supernatants from doxycycline-induced Hibit16 reporter cells were immunoprecipitated with anti-core antibody-conjugated beads and analyzed using antibodies targeting the core/precore region together with the HiBiT blotting system.

### Cell viability

Cell viability after palovarotene treatment was assessed using the CCK-8 assay (Cell Counting Kit-8, MedChemExpress LLC, Monmouth Junction, NJ, USA) in HepG2-Rccc1a, HepAD38, HepG2-hNTCP-2B1, and primary human hepatocyte cultures under the same concentration ranges used for antiviral evaluation.

## Results

### Engineering a replication-competent, cccDNA-linked reporter in HepaRG (HepaRG-Hibit16)

The HBV genome is small, but highly intricate and complex. The structure of the HBV genome presents a significant challenge to modifications, as the direct insertion of full-length reporter gene sequences into the HBV genome severely impacts the virus’s replication capacity. In this study, a small fragment of split NanoLuc (HiBiT/LgBiT), HiBiT, was integrated into the HBV genome as a reporter gene for cccDNA. The length of HiBiT coding sequence is only 33 bp, theoretically causing minimal interference with HBV, allowing the virus to replicate and produce cccDNA.

**Figure 1.**
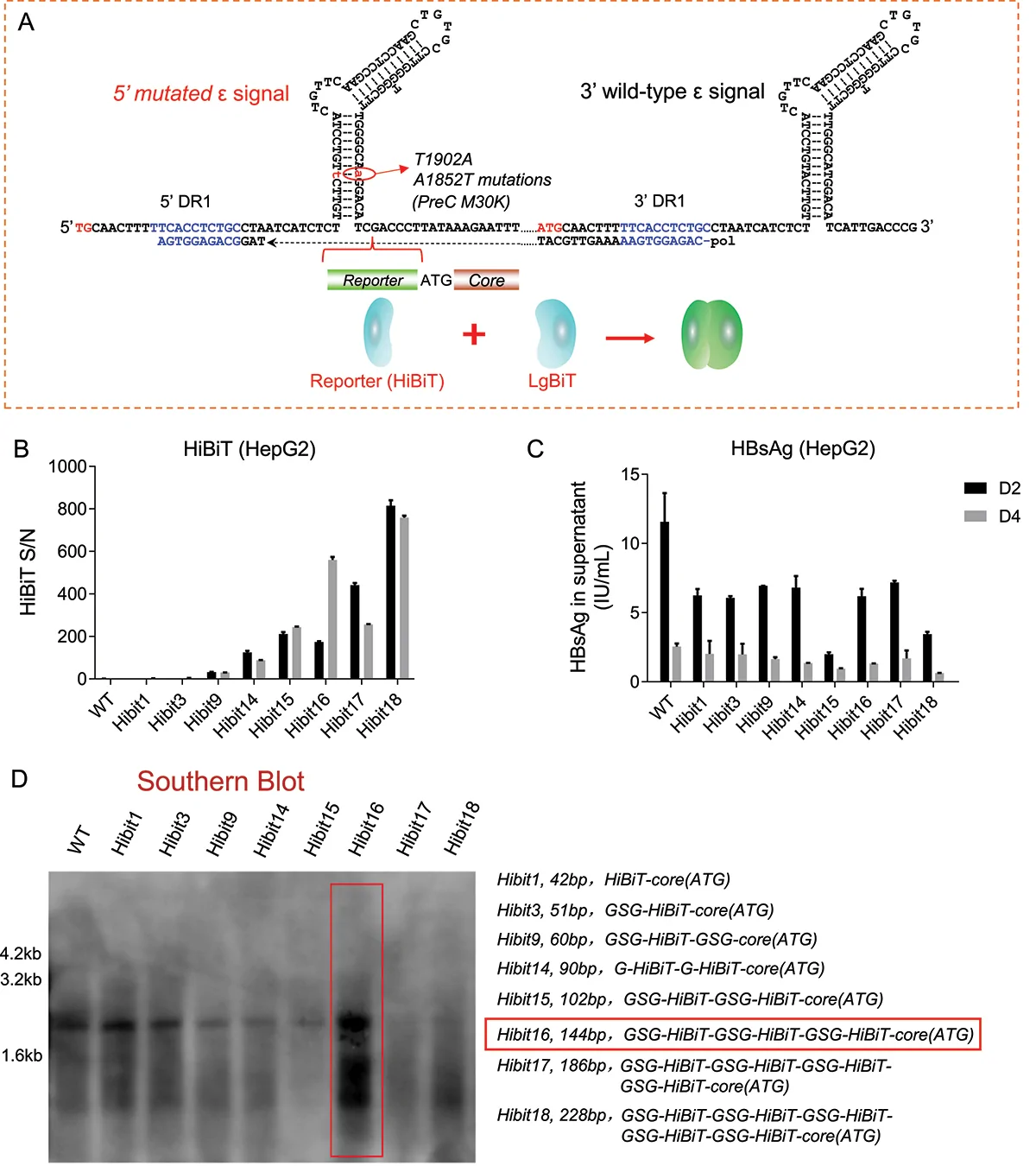
Design and comparison of replicable HBV variants with integrated differing copies of the HiBiT sequence (A) The design of HBV variant of which the expression of the reporter gene HiBiT is dependent on the formation of cccDNA. Expression of HiBiT tags (B) And antigens (C) In HBV variant that integrated with different optimized HiBiT sequences. (D) Comparison of the replication capabilities of HBV variants.

The design of HiBiT integration into the HBV genome, as shown in Figure 1(A) and Supplementary figure S1A, places the HiBiT fragment at the C-terminal of the pre-core open reading frame (ORF). The HiBiT tag, secreted with HBeAg into the extracellular medium, can be detectable by mixing cell culture supernatant with two components from the detection kit and reading the luminescence value. The unique design of the epsilon sequence makes HiBiT translation dependent on the formation of HBV cccDNA [14]: the start codon of the pre-core domain at the 5’ end of the 1.1-fold HBV genome retains only “TG,” and the start codon at position 1901 is mutated to AAG, rendering RNA transcribed from this sequence incapable of translating the HiBiT tag. However, the start codon of the pre-core domain at the 3’ end is intact, allowing for the repair of the pre-core domain start codon during HBV cccDNA formation, enabling RNA transcribed from HBV cccDNA to translate the HiBiT signal. We found that the signal from direct HiBiT integration was weak, so we experimented with 1–5 copies of HiBiT in tandem and tried different linker peptide sequences. Considering the HiBiT signal, viral protein expression, and replication capability of these eight mutations (Figure 1(B–D)), we identified variant Hibit16 as the optimal one, producing a strong HiBiT signal with minimal interference on HBV itself.

**Figure 2.**
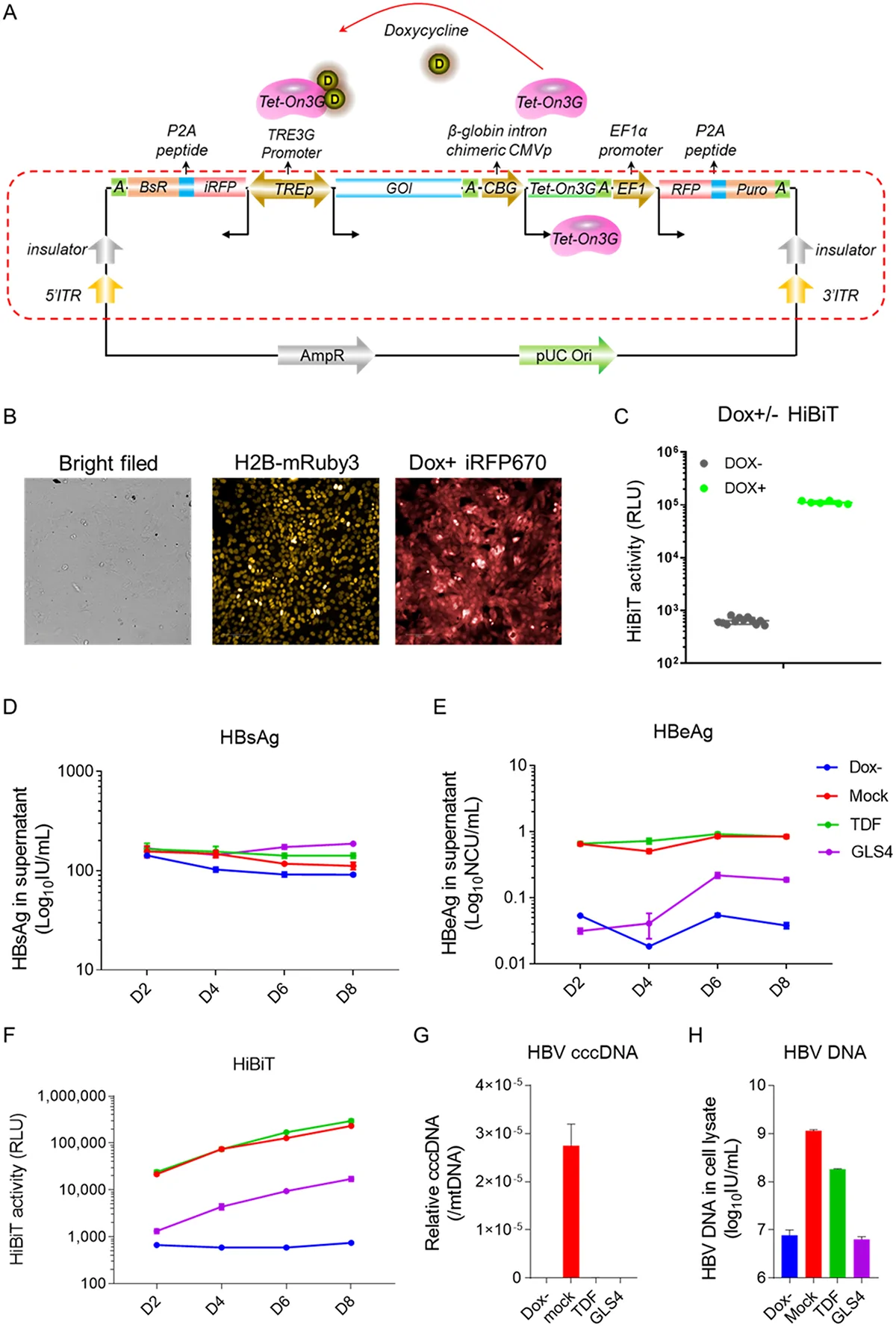
Construction of the HepaRG-Hibit16 cell model with stably integrated HBV variant Hibit16 (A) Schematic diagram of the Hibit16 plasmid vector. (B-C) Morphology, fluorescence, and HiBiT signal of HepaRG-Hibit16 cells. (D-H) Virological indicators and HiBiT signal responses of HepaRG-Hibit16 cells to HBV inhibitors. D/Dox: Doxycycline, A: poly(A) tail, BsR: Blasticidin S-resistance gene, iRFP: near-infrared fluorescent protein, GOI: gene of interest, RFP: mRuby3, Puro: puromycin.

Using the PiggyBac transposon system, Hibit16 was integrated into the genome of HepaRG cells, and stable cell line HepaRG-Hibit16 was selected using the fluorescent marker RFP (mRuby3) and resistance to Puromycin (Figure 2(A)). RFP is continuously expressed in HepaRG-Hibit16 cells, localized in the nucleus, while iRFP670 and the target gene HBV expressed under the induction of Doxycycline (Figure 2(B,C)). The initial formation of cccDNA are regulated by the Tet-on expression control system. Under Doxycycline induction, HBV replication, HiBiT signal, and the expression of HBsAg and HBeAg can be detected. To detect HiBiT sequence within cccDNA directly, cccDNA-enriched PCR followed by Sanger sequencing was performed. We detected the expected HiBiT sequence in the cccDNA-derived amplicon from Hibit16 reporter cells (Supplementary figure S1B).

The model’s response to HBV inhibitors was also preliminarily assessed by intervening with Tenofovir Disoproxil Fumarate (TDF) and Morphothiadin (GLS4) [26] while inducing with Doxycycline. TDF and GLS4 could inhibit the formation of HBV cccDNA in this model as expected. GLS4 also significantly suppressed the expression of HiBiT (Figure 2(D–H)). However, TDF did not proportionally suppress total extracellular HiBiT activity. To clarify this discrepancy, we immunoprecipitated secreted core/precore-related products and detected HiBiT-containing proteins by HiBiT blotting. These experiments revealed an expected HiBiT-precore/HBe-related product together with an additional lower-molecular-weight HiBiT-containing species (Supplementary figure S2). Inspection of the 5’ epsilon/proximal HiBiT region identified candidate near-cognate/non-AUG initiation codons upstream of the HiBiT sequence. Because non-AUG initiation, particularly from CUG and other near-cognate codons, can occur in eukaryotic mRNAs [27], we interpret the residual TDF-insensitive HiBiT signal as a cccDNA-independent component derived from doxycycline-induced HBV transcripts. Accordingly, we now describe HepaRG-Hibit16 as a cccDNA-linked reporter model, rather than a direct or exclusive cccDNA quantification system.

### Construction of HiBiT-tagged HBV rcccDNA reporter model HepG2-Rccc1a

The HBV rcccDNA reporter model employs Cre-Lox recombination to synthesize rcccDNA within cells rapidly, achieving higher efficiency than cccDNA produced through HBV replication [17,28]. The model introduces HiBiT, a marker whose expression depends on the presence of rcccDNA, serving as an alternative indicator for rcccDNA detection.

**Figure 3.**
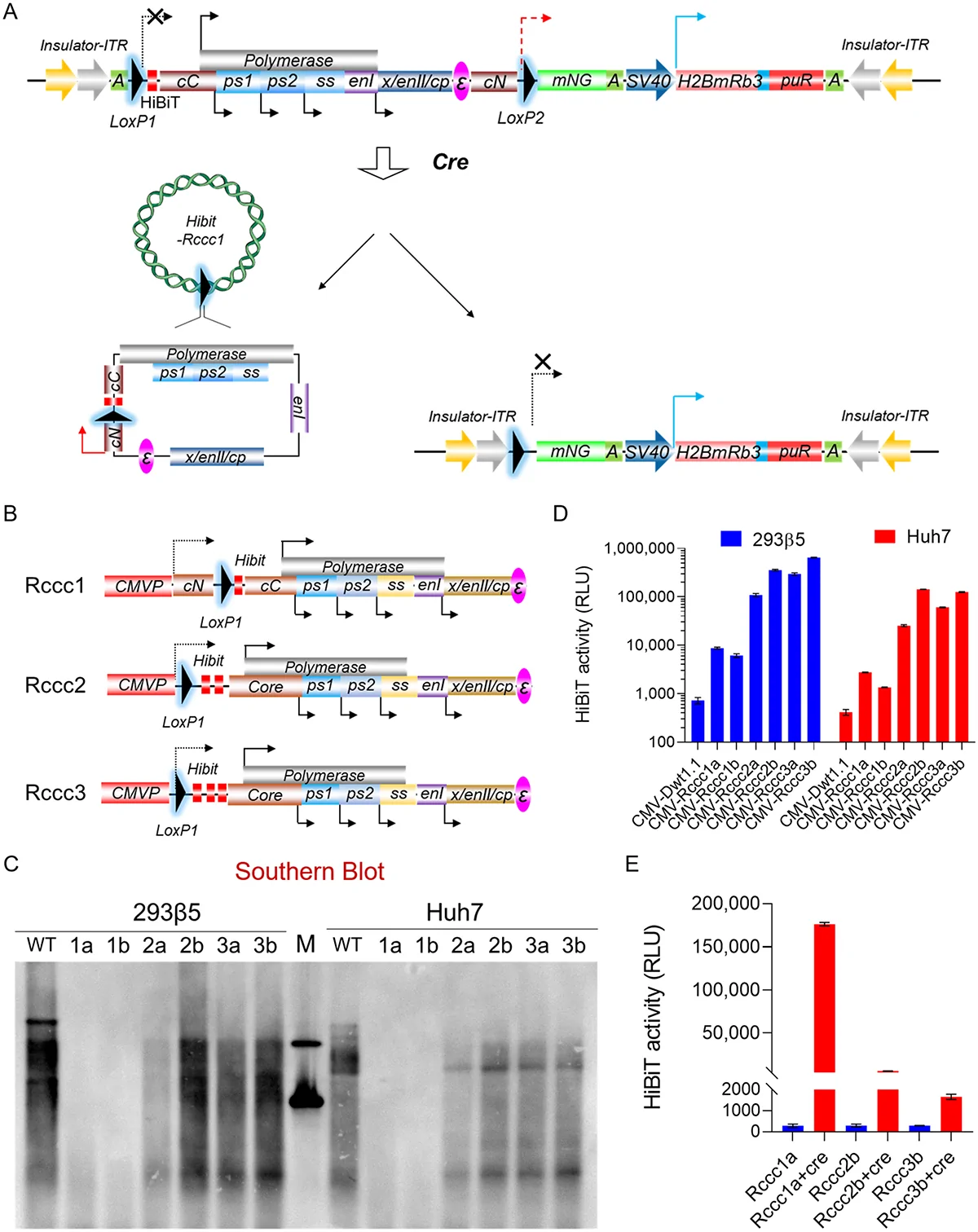
Design of the HBV rcccDNA reporter model indicated by luciferase (A) Schematic diagram of HBV rcccDNA formation mediated by Cre protein. (B) Three different HiBiT tag insertion methods. (C-D) Comparison of replication capabilities and HiBiT signals of six different variants with exogenous promoters. (E) the ability of three variants to produce HiBiT signals dependent on rcccDNA in the absence of exogenous promoters. A: poly(A) tail, LoxP: LoxP sites, cC: C-terminal of core ORF, ps1: pre S1 ORF, ps2: pre S2 ORF, ss: s ORF, cN: N-terminal of core ORF, mNG: mNeongreen, SV40: SV40 promoter sequence, H2BmRb3: sequence of nuclear localized mRuby3, puR: puromycin.

The method for generating rcccDNA based on Cre recombinase involves attaching LoxP sequences to both ends of the HBV genome. Upon the expression of Cre recombinase, the sequences flanking the LoxP sites are connected, and the sequences between the LoxP sites cyclize to form a double-stranded closed circular DNA structure similar to HBV cccDNA, as shown in Figure 3(A). The integrated HBV sequence lacks exogenous promoters, and the reporter gene utilizes HBV’s endogenous promoters, allowing translation only upon the formation of rcccDNA. We designed mutations inserting 2 and 3 HiBiT units at the C end of pre-core, in addition to a mutation inserting a single HiBiT sequence into the apical loop region of the core protein, and evaluated the effects of two LoxP sequences (a: ataacttcgtataatgtatgctatacgaagttat; b: ataacttcgtatagcatacattatacgaagttat). Six combinations of external gene insertion methods and two distinct LoxP sequences were assessed for replication capacity in 293β5 and Huh7 cells under conditions with exogenous promoters (Figure 3(B) and Supplementary figure S3). Results shown in Figure 3(C) indicate that 2a, 2b, 3a, and 3b exhibited higher replication abilities than 1a and 1b in both cell types; mutations using the LoxPb sequence showed stronger replication capacity than those using the LoxPa sequence. Figure 3(D) reports HiBiT signals produced directly from the CMV-driven plasmid, with HiBiT tags placed at the C end of pre-core, achieving higher HiBiT expression levels. Considering HiBiT expression, we continued with 1a, 2b, and 3b for subsequent work, removing their exogenous promoters and connecting LoxPa or LoxPb at both ends of the HBV sequence to obtain HibitRccc1a, HibitRccc2b, and HibitRccc3b. Figure 3(E) shows the HiBiT expression following co-expression with Cre recombinase after removal of exogenous promoters, where all three modified viruses expressed detectable HiBiT signals when co-expressed with Cre recombinase, with HibitRccc1a exhibiting significantly higher HiBiT signals than the others.

**Figure 4.**
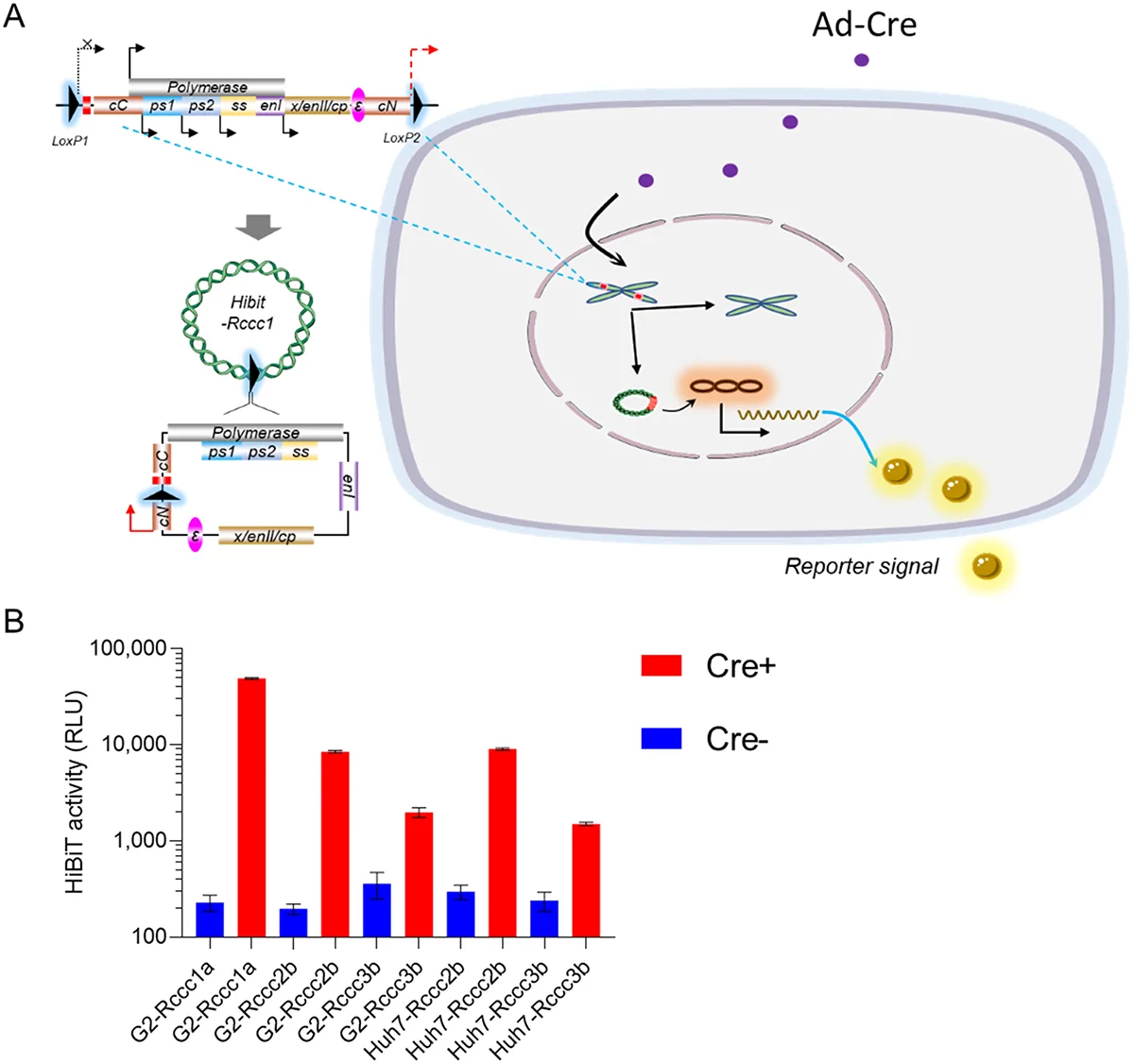
Construction of the rcccDNA reporter model cell lines (A) Schematic diagram of the cell line construction method. (B) HiBiT expression in five rcccDNA reporter model cell lines after treatment with Ad-Cre.

To prevent the expression of Cre recombinase from affecting the number of HBV copies integrated into the cell genome during the construction of cell lines, the HBV sequence was integrated into the cell genome without integrating the Cre protein coding sequence. Then expressing Cre recombinase by infecting cells with adenovirus (Figure 4(A)). Vectors of HibitRccc1a, HibitRccc2b, and HibitRccc3b contained selection markers of red fluorescent protein mRuby3 and puromycin resistance gene, and we integrated HibitRccc1a, HibitRccc2b, and HibitRccc3b into Huh7 and HepG2 cells using the PiggyBac transposon system, selecting stable integrated cell lines through puromycin resistance and red fluorescence. After obtaining the integrated cell lines, they were treated with Ad-Cre to induce the formation of rcccDNA and expression of HiBiT. Ad-Cre infection enabled these cell lines to secrete proteins tagged with HiBiT, and HepG2 cells integrating HibitRccc1a, named HepG2-Rccc1a, exhibited significantly higher HiBiT signals than other cell lines (Figure 4(B)). PCR and Sanger sequencing across the recombination junction confirmed the expected LoxP-HiBiT-core-N/core-C arrangement in the recombined rcccDNA product (Supplementary figure S4).

### High-throughput screening nominates concordant hits across models

**Figure 5.**
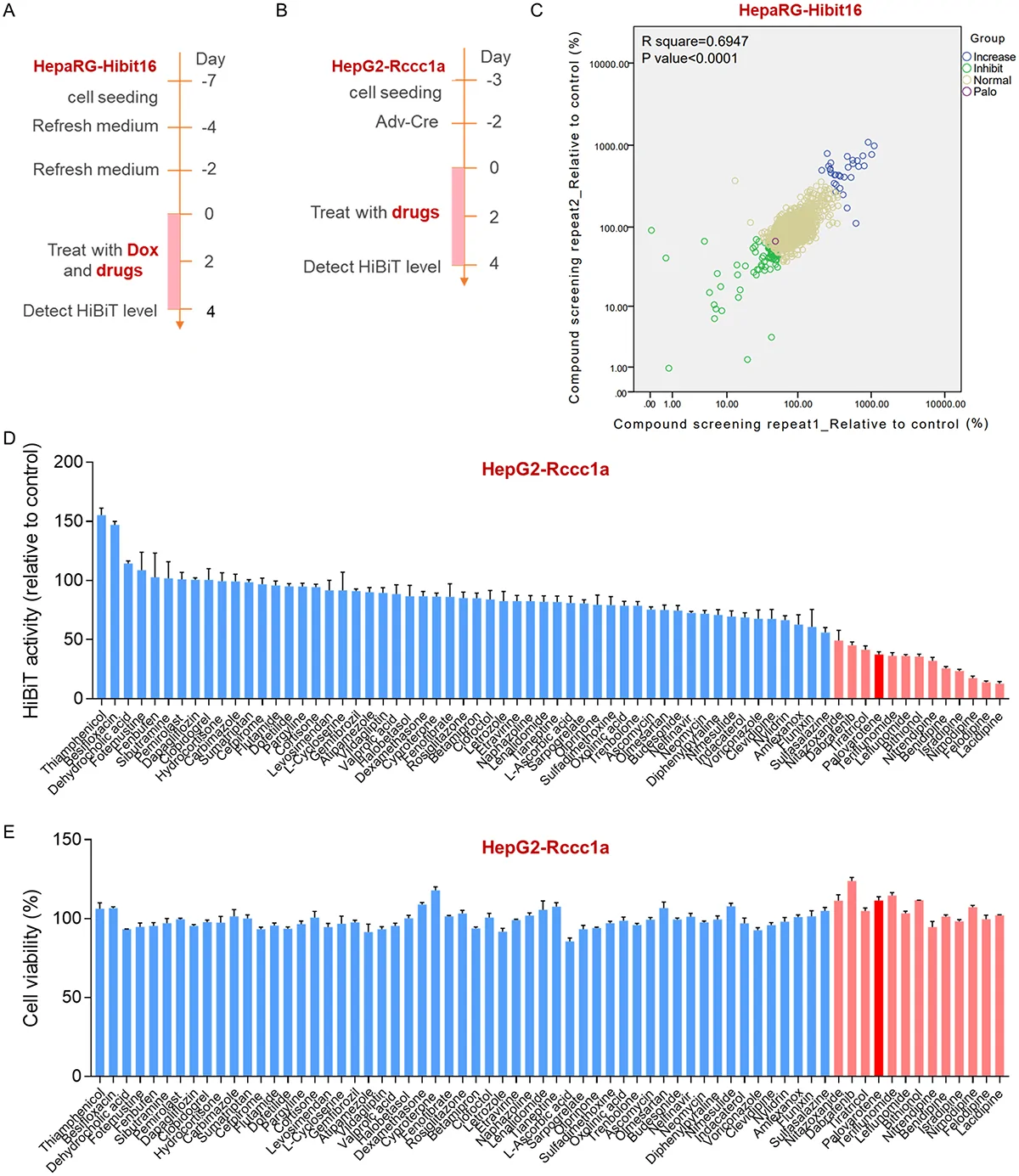
HepaRG-Hibi16 and HepG2-Rccc1a used for drug screening (A) The drug screening process using the HepaRG-Hibit16 model. (B) The drug screening process using the HepG2-Rccc1a model. (C) Screening results of 1,403 drugs in the HepaRG-Hibit16 model. (D-E) Further validation of the efficacy and safety of compounds selected using the HepaRG-Hibit16 model in the HepG2-Rccc1a model.

We next assessed whether the two reporter models could be deployed for practical high-throughput screening. We assessed 1,403 FDA-approved drugs (Supplementary table 1) with these two cell models, screening for compounds that inhibit the HiBiT signal (Figure 5). The drug screening process for both models is depicted in Figure 5(A,B). Initially, all drugs were evaluated for their functionality and cytotoxicity in the RG-Hibi16 cell model, selecting drugs with an inhibition rate greater than 50% and post-intervention cell viability greater than 95% (Figure 5(C)). For further assessment in the HepG2-Rccc1a model (Figure 5(D,E)), 13 drugs were identified that met this criterion in both models. Among these, Nitazoxanide has been reported to inhibit the interaction between HBx and DDB1, thereby reducing viral transcription [29], indicating that the HepaRG-Hibi16 and HepG2-Rccc1a models can be used to screen for drugs that have a virological level of inhibition against HBV. Additionally, six of the drugs are calcium channel blockers, which may inhibit HBV through effects on the Ca2+ pathway; the drug Palovarotene, an agonist of the retinoic acid receptor gamma (RAR-γ), may inhibit the transcription from HBV cccDNA as a template. The drugs also include the anticancer drug Dabrafenib, immunosuppressive drugs Leflunomide and Teriflunomide, the thyroid hormone analogue Tiratricol, and the antibacterial agent Bithionol, none of which have previously been reported for the treatment of chronic hepatitis B. These data establish feasibility of using paired HiBiT-based reporter systems for efficient hit triage and prioritization of candidate cccDNA modulators.

### Palovarotene reduces HBV markers *in vitro* as an exemplar concordant hit

**Figure 6.**
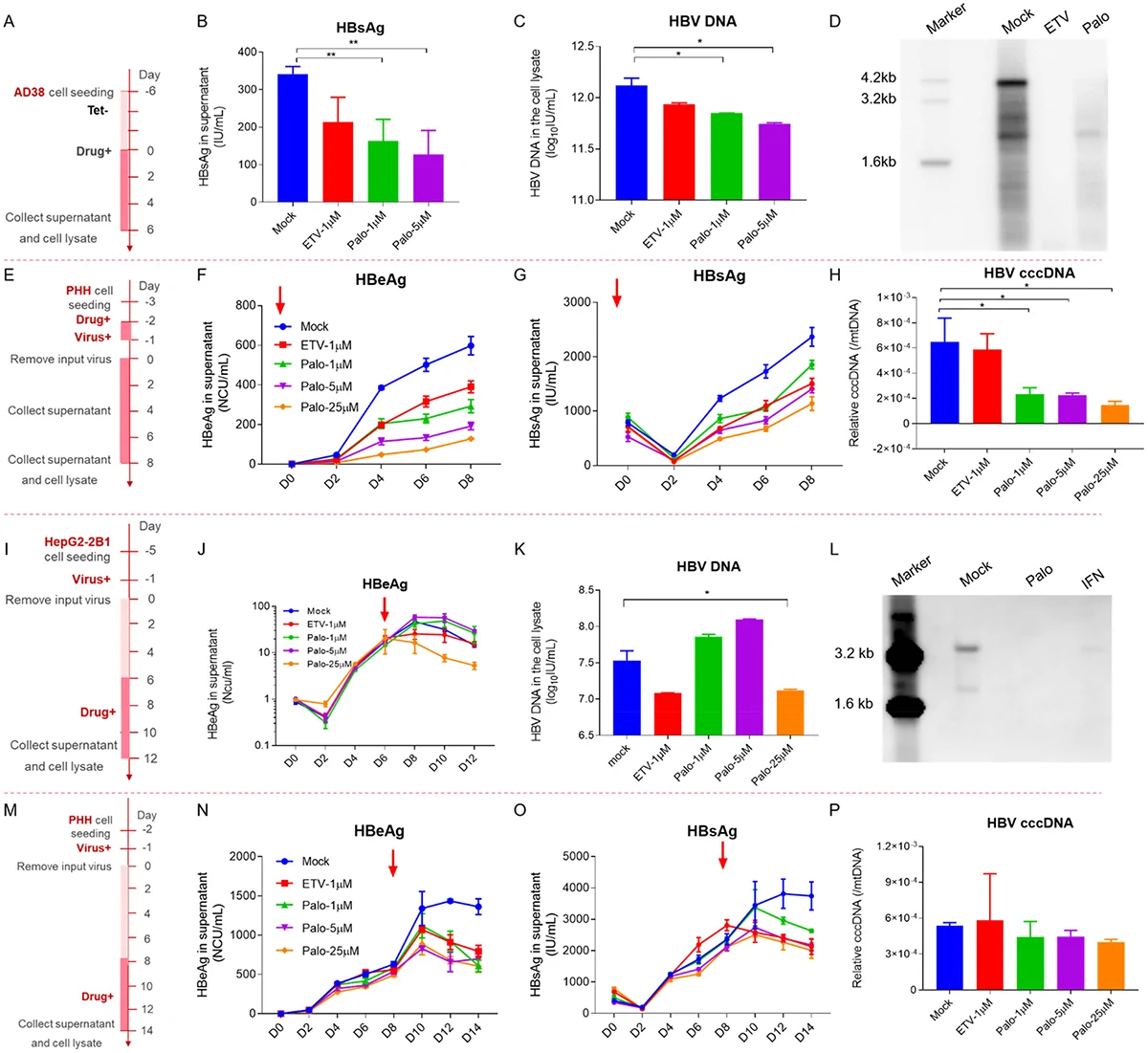
Evaluating the inhibitory effects of palovarotene on HBV in different *in vitro* study models (A-D) Assessment of Palovarotene’s inhibitory effects on HBV antigens and DNA in the HepAD38 cell model. (E-H) Assessment of the inhibitory effects of pre-infection Palovarotene treatment on HBV antigens and cccDNA in the PHH model. (I-L) Evaluation of the inhibitory effects of post-infection Palovarotene treatment on HBV antigens and cccDNA in the HepG2-hNTCP-2B1 model. (M-P) Evaluation of the inhibitory effects of post-infection Palovarotene treatment on HBV antigens and cccDNA in the PHH model.

Palovarotene is an agonist of the retinoic acid receptor gamma (RAR-γ) approved by the U.S. Food and Drug Administration for the treatment of Fibrodysplasia Ossificans Progressiva (FOP). This study, based on the cccDNA reporter model, discovered that Palovarotene inhibits cccDNA-linked HiBiT signal, prompting further validation of Palovarotene’s function in other HBV research models. Figure 6(A–D) evaluate the inhibitory effects of Palovarotene on HBV in the hepatocellular carcinoma cell line HepAD38, which stably integrates the HBV genome sequence. In these cells, Palovarotene not only reduces the expression level of the surface antigen HBsAg but also decreases HBV replication. The impact of Palovarotene on HBV was also assessed in HepG2-hNTCP-2B1 [21] and primary hepatocyte infection models. Figure 6(E–H) show that treating primary hepatocytes with various concentrations of Palovarotene (1 μM, 5 μM, and 25 μM) 24 hours before HBV infection significantly inhibits HBV infection of PHHs, significantly reducing HBV antigens (HBsAg and HBeAg), and HBV replication. In addition to pre-treatment, this study also evaluated the effect of administering Palovarotene after infection to assess the compound’s therapeutic potential. Figure 6(I–L) depict the intervention with Palovarotene in HepG2-hNTCP-2B1 cells 6 days post-HBV infection, showing that 25 μM of Palovarotene effectively reduces the expression level of the HBV antigen HBeAg and has a significant inhibitory effect on HBV replication. Figure 6(M–P) demonstrate the intervention with Palovarotene in primary hepatocytes 8 days post-HBV infection, where all three concentrations of Palovarotene (1 μM, 5 μM, and 25 μM) effectively reduce the expression levels of HBV antigens HBsAg and HBeAg. Thus, Palovarotene effectively reduces virological levels of HBV, whether administered before or after infection.

## Discussion

In this work, we address a practical bottleneck in HBV cure-oriented research: the lack of scalable assays that report on cccDNA activity in a way that is suitable for high-throughput discovery. We engineered two complementary hepatocyte reporter models that link a sensitive split-luciferase HiBiT output to cccDNA (or rcccDNA) transcriptional activity, and we demonstrate that both models support compound testing under multiwell conditions. The key design principle is complementarity. HepaRG-HiBiT16 couples a replication-competent HBV reporter to a secreted luminescent readout in hepatocyte-like cells, thereby retaining steps upstream of cccDNA establishment and supporting interrogation of compounds that affect replication-linked cccDNA expression. In contrast, the HepG2-Rccc1a Cre/Lox system rapidly generates rcccDNA episomes and provides a controllable, orthogonal readout that is less dependent on viral entry and replication. Used together, these models allow concordance-based triage that can reduce false positives driven by cell-line-specific toxicity, pathway idiosyncrasies, or reporter artifacts, while retaining throughput.

A pilot screen of 1,403 FDA-approved compounds illustrates the value of this dual-model approach. The 13 concordant hits provide a focused starting point for downstream mechanistic studies of host pathways that modulate cccDNA transcription. We selected palovarotene as an exemplar concordant hit. Across several hepatocyte-based *in vitro* HBV systems, palovarotene reduced viral antigens and nucleic acids, supporting its activity on HBV markers in cell culture.

Using alternative indicators for HBV cccDNA to increase detection efficiency and reduce costs for high-throughput drug screening is a viable strategy for developing drugs against HBV cccDNA. Existing HBV cccDNA reporter models with higher throughput include those developed by Haitao Guo et al. using HBeAg or HA as alternative indicators for cccDNA detection [13,14], and models developed by Min Wu et al. using DNA recombination to express HBsAg as an indicator for rcccDNA [15]. These cell models use antigens HBeAg, HA and HBsAg as reporter genes for cccDNA, reducing detection difficulty and increasing throughput compared to direct cccDNA detection. While antigen detection involves more steps and time, luciferase detection is simpler, quicker, and allows for higher throughput screening. Although Feng Li and colleagues developed a reporter model using luciferase as a surrogate indicator for rcccDNA, simplifying the detection process, the initial synthesis of rcccDNA was carried out in prokaryotic cells before transfection into hepatoma cells, which complicates the operational steps [16]. Nakaya et.al established HBV-HiBiT-PreS2 system, their system is valuable for monitoring HBV replication and HBsAg/subviral-particle-related secretion, further demonstrated the value of a short HiBiT tag [19].

This study aims to construct stable and efficient cccDNA-linked reporter models for drug screening targeting cccDNA. Two strategies were adopted to construct cccDNA drug screening models: one strategy is inserting a tri-copy HiBiT tag between the pre-core and core sequences, removing the start codon of pre-core in the N-terminal of 1.1-fold HBV genome, thus allowing the HiBiT-tagged protein to be translated from cccDNA produced during HBV replication; the other strategy involves inserting a HiBiT tag in the middle of the core sequence and coupling LoxP sequences to both ends of the 1-fold HBV genome sequence, enabling double-stranded HBV DNA to recombine within the cell to form rcccDNA. Since no exogenous promoter can initiate the expression of the HiBiT-tagged protein, HiBiT can only start translation using the core’s promoter after the formation of rcccDNA. In the former strategy, cccDNA sources from the virus’s replication process, mimicking the formation of cccDNA in patients, whereas in the latter strategy rcccDNA sources from Cre recombinase-mediated DNA recombination, with a high efficiency of rcccDNA formation. Based on these two strategies, we constructed the HepaRG-Hibit16 and HepG2-Rccc1a cell lines. Both cell lines secreted HiBiT signal related to cccDNA levels, which simplifies operations and shortens detection time.

In HepaRG-Hibit16 cells, the replication and expression of virus are controlled by Doxycycline, making this cell model suitable for screening inhibitors that interfere with cccDNA formation. Preliminary assessments of this model showed that it could be used to screen inhibitors like GLS4 that inhibit cccDNA formation by regulating core assembly, with HiBiT correlation to cccDNA superior to other antigens’ correlation with cccDNA. The main advantage of this model lies in the simplicity and speed of HiBiT detection, thus providing high throughput for compound screening.

This study also constructed a reporter model that directly synthesizes rcccDNA through DNA recombination for screening inhibitors that clear or silence cccDNA. The work of Min Wu and others proved that rcccDNA obtained through DNA recombination can bind histones to form a minichromosome, similar to wild-type cccDNA, hence rcccDNA reporter models can be used to screen cccDNA inhibitors. According to Min Wu’s study, expressing Cre recombinase through adenovirus infection can fully form rcccDNA from HBV DNA integrated into the cell genome within three days, indicating high efficiency of rcccDNA production [15]. This study constructed a rcccDNA reporter model using HiBiT as a detection indicator, which, compared to HepaRG-Hibit16, accelerates rcccDNA formation, shortening cell culture and drug screening periods and simplifying operational steps; compared to rcccDNA reporter models using HBsAg or other antigens as detection indicators, HiBiT detection offers significant advantages. Although Feng Li and others reported a reporter model using GLuc as a surrogate indicator for rcccDNA, synthesizing mcHBV-GLuc cccDNA in bacteria and then transfecting into hepatoma cells is costly and challenging for high-throughput operations [14]. Constructing integrated cell lines that synthesize rcccDNA directly within cells is more convenient, stable, and efficient. The HepG2-Rccc1a cell line constructed based on this strategy can form rcccDNA and secrete HiBiT, which is more suitable for high-throughput screening.

Through the cccDNA-linked reporter model, this study identified Palovarotene as having an inhibitory effect on cccDNA-linked HiBiT signal. Furthermore, Palovarotene’s inhibitory action on HBV was validated in the HBV replication model HepAD38 cell model, as well as in HepG2-hNTCP-2B1 infection model and primary hepatocyte infection models, confirming Palovarotene can inhibit HBV infection and reduce various HBV markers. Baocun Li [30] and Shirin Nkongolo [31] reported in 2018 and 2019, respectively, that RAR agonists Tazarotene and Am80 could inhibit transcription templated by HBV cccDNA. Combining these reports with the results of this study, Palovarotene’s inhibition of HBV may be through inhibiting HBV replication and transcription templated by HBV cccDNA. Nevertheless, the detail effects and molecular mechanisms underlying these biological effects of palovarotene remain to be elucidated in future investigations.

This study has limitations. First, luminescent HiBiT secretion is an indirect proxy for cccDNA transcription and can be influenced by post-transcriptional effects; accordingly, orthogonal virologic readouts remain essential for hit validation. Second, HiBiT luminescent signals may also arise through cccDNA-independent pathways, such as translation initiated from non-AUG start codons, which could introduce background interference in our reporter assay. Third, our screen was limited to an FDA-approved library and to the two model contexts described here; broader chemical space and additional infection-derived systems may capture complementary mechanisms. Fourth, we did not validate the functional activity of palovarotene in physiologically relevant animal models, such as human liver chimeric mice, which are required to faithfully recapitulate human HBV infection and cccDNA biology.

Despite these limitations, the paired reporter strategy provides an accessible and scalable workflow for hepatology laboratories to identify and prioritize candidate cccDNA transcription modulators. By combining a replication-linked cccDNA model with a rapid rcccDNA model, the platform can accelerate early-stage discovery and enable more efficient allocation of resources to the most reproducible candidates for deeper mechanistic and translational evaluation.

In summary, HepaRG-Hibit16 and HepG2-Rccc1a establish a paired, screening-compatible workflow for identifying candidate modulators of cccDNA-linked transcriptional output. The value of the platform lies in rapid, sensitive triage and cross-model prioritization, followed by orthogonal virological validation in infection-derived systems.

## Supplementary Material

Supplementary table 1.xlsx

Supplementary.docx

## Data Availability

The Data generated during the study is available at repository “figshare” at https://doi.org/10.6084/m9.figshare.31819390.v2 [32]
