# Supplementary material for: Dual HBV cccDNA-linked HiBiT reporter hepatocyte models for screening of candidate cccDNA modulators
Source: Virulence. 2026 Sep 4;17(1):2728526. doi: 10.1080/21505594.2026.2728526 (PMC13556949; doi:10.1080/21505594.2026.2728526)
Supplement: Supplementary file 1 [file KVIR_A_2728526_SM1494.docx]

**Supplementary figure S1 Hibit16 design and detection of HiBiT sequence in cccDNA-enriched DNA.** (A) Nucleotide-level design of the Hibit16 reporter region. (B) Sanger sequencing of a cccDNA-enriched PCR amplicon showing the expected HiBiT-containing sequence.

**Supplementary figure S2**  **Immunoprecipitation/HiBiT blot analysis of secreted HiBiT-containing products.** Supernatants from doxycycline-induced HepaRG-Hibit16 reporter cells were immunoprecipitated with anti-core beads and detected using antibodies targeting core/precore regions or the HiBiT blotting system. Two HiBiT-containing species were observed: a product compatible with the expected HiBiT-precore/HBe-related species and a lower-molecular-weight HiBiT-containing species.

**Supplementary figure S3 Nucleotide-level designs of** (A) **Rccc1a,** (B) **Rccc2b, and** (C) **Rccc3b reporter.**

**Supplementary figure S4 Rccc1a circularization design and sequencing validation.** (A) Schematic representation of Cre/Lox-mediated circularization. (B) Sanger sequencing confirmed the expected rcccDNA recombination junction containing the LoxP and HiBiT sequences between the core-N and core-C regions.

**Supplementary figure S5 CCK-8 viability assays after palovarotene treatment.** Cell viability was assessed in (A) HepAD38, (B) HepG2-hNTCP-2B1, and (C) primary human hepatocytes under the concentration ranges used for antiviral evaluation.
